# Detection and Quantification of *Legionella pneumophila* from Water Systems in Kuwait Residential Facilities

**DOI:** 10.1155/2012/138389

**Published:** 2012-07-24

**Authors:** Qadreyah A. Al-Matawah, Sameer F. Al-Zenki, Jafer A. Qasem, Tahani E. Al-Waalan, Ahmed H. Ben Heji

**Affiliations:** ^1^Biotechnology Department, Kuwait Institute for Scientific Research, Safat 13109, Kuwait; ^2^Department of Applied Medical Sciences, College of Health Sciences, The Public Authority for Applied Education and Training, Safat 13092, Kuwait

## Abstract

The prevalence of *Legionella pneumophilia* in water systems of residential facilities in Kuwait was performed during the period from November 2007 to November 2011. A total of 204 water samples collected from faucets and showerheads in bathrooms (*n* = 82), taps in kitchens (*n* = 51), and water tanks (*n* = 71), from different locations of residential facilities in Kuwait were screened for *Legionella pneumophila* by the standard culture method and by real time polymerase chain reaction (RT-PCR). Out of the 204 samples, 89 (43.6%) samples were positive for *Legionella* spp., 48 (23.5%) samples were detected by the standard culture method, and 85 (41.7%) were detected by RT-PCR. Of the culture positive *Legionella* samples, counts ranged between 10 to 2250 CFU/L. Serological typing of 48 *Legionella* isolates revealed that 6 (12.5%) of these isolates belonged to *Legionella pneumophila* serogroup 1, 37 (77.1%) isolates to *Legionella pneumophila* serogroup 3, and 1 isolate each (2.1%) belonged to serogroups 4, 7, and 10. The minimum inhibitory concentration (MICs) of the 46 environmental *L. pneumophila* isolates against the 10 antimicrobials commonly used for *Legionella* infection treatments were determined. Rifampicin was found to be the most active against *L. pneumophila* serogroups isolates *in vitro*.

## 1. Introduction

Outbreaks of Legionnaires' disease have been worldwide traced to a wide variety of environmental water sources such as cooling towers, hot tubs, showerheads, whirlpools and spas, and public fountains [1, 2]. *Legionella pneumophila* serogroup 1 is responsible for up to 80% of Legionnaires' disease reported cases [3, 4]. The potential health risk of *Legionella* to humans is theoretically associated with cells densities above 10^4^ to 10^5^ CFU per liter of water [5, 6]. 

Commonly used method for environmental surveillance of *Legionella* is the standard culture technique [7, 8]. Although the standard culture method allows the isolation and the quantification of *Legionella* from the environment, it does have its limitations: it requires selective media and prolonged incubation periods; bacterial loss can occur during the concentration stage followed by decontamination with heat or acid; interference of background organisms with *Legionella *growth may lead to an underestimation of the real number of *Legionella* present in the sample; *Legionella* spp. may enter a viable but noncultivable state, making it difficult to culture from water samples [9]. 

Recently, rapid and sensitive alternative methods have been found to be attractive alternatives to the conventional culture method for the detection of slow-growing and fastidious bacteria such as *Legionella*. These methods are PCR-based methods for the detection and quantification of *Legionella *in water that has been used primarily against the 5S and 16S rRNA genes and against the macrophage infectivity potentiator (*mip*) gene of *L. pneumophila *[10–15]. A real-time-PCR-based method for rapid detection and quantification of *Legionella* in water samples have been developed [15–17]. Several commercial real-time PCR kits are now available, and the main differences among these kits are based on the degrees of standardization of the three critical steps: DNA extraction, PCR preparation, and data analysis. However, all the PCR-based methods lack the ability to discriminate between living and nonliving (noninfectious) *Legionella *cells.

Despite the fact that environmental *Legionella* monitoring is recommended in several countries, in Kuwait, environmental monitoring of *Legionella* is not conducted and no active surveillance program exists. Moreover, research related to *Legionella* is scarce [18–20]. Furthermore, no reports are available on the current status of the prevalence of Legionnaires' disease and associated cases in Kuwait. However, annual reports presented by the Ministry of Planning show that the death of a percentage of the population (27.6/10^4^ of population) is due to respiratory diseases without specifying the etiological agent [21]. 

Due to the following factors, it is likely that the prevalence of *Legionella* is underestimated; Kuwait's a hot temperate climate; the absence of water safety regulations for *Legionella* monitoring and decontamination; and the increased use of cooling towers within recreational and health care facilities may increase the risk of legionellosis. In addition, water temperatures in water tanks during the summer in residential compounds may be favorable for *Legionella *multiplication (50°C). Owing to the possibility of environmental exposure to *Legionella*, this is the first study aimed at determining the prevalence of *Legionella *in selected residential facilities in Kuwait.

## 2. Material and Methods

### 2.1. Water Samples

A total of 204 samples were collected from November 2007 to November 2011. Water samples were obtained from faucets of wash basins and showerheads in bathrooms, taps (*n* = 82) from kitchens (*n* = 51) and cold/hot water tanks (*n* = 71), from different residential sites in the State of Kuwait. Water samples (1 L) were collected in a sterile 2 L plastic bottle containing 1mL of 0.1 N sodium thiosulfate to neutralize chlorine disinfectant.

### 2.2. Sample Concentration

The 1 L sample was filter concentrated in a biological safety cabinet by pouring the sample into a sterile 47 mm filter funnel assembly containing a 0.2 *μ*m polycarbonate filter (Fisher Scientific, 3970 Johns Creek Ct., Suite 500, Suwanee, GA 30024). When the sample had passed through the filter, the filter was removed aseptically from the holder with sterile filter forceps, folded to the outside, and placed into a sterile, 50 mL centrifuge tube containing 10 mL of sterile water. Then, the centrifuge tube was vortexed for one minute to free bacteria and organic material from the filter and centrifuged at 3000 ×g ± 100 ×g for 30 min ± 1 min. Using a sterile graduated pipette aseptically, all but 1 mL of supernatant was carefully removed. The deposit was resuspended by vortex mixing. This constitutes the final concentrate that was stored in screw-capped containers at 6°C ± 2°C in the dark.

### 2.3. Standard Culture Method

From the final concentrate, 0.1 mL was inoculated onto buffered charcoal-yeast extract agar base (code CM0655; Oxoid; UK) with MWY selective supplement (code SR0118; Oxoid; UK), and 0.01 mL was inoculated onto BCYE agar with BMPA selective supplement (code SR0111; Oxoid; UK). Subsequently, 0.2 mL was heat treated at 50°C for 30 min, and 0.1 mL and 0.01 mL was inoculated onto MWY agar. Additionally, 0.2 mL of sample were acid treated in equal volume of HCl-KCl acid buffer (pH 2.2) for 5 min, and 0.1 mL was inoculated onto BMPA agar. All plates were incubated at 35°C in CO_2_ incubator. The plates were examined after 72 h to 96 h (4 to 7 d) incubation [22]. Suspect colonies were aseptically picked and streaked onto BCYE agar plate without L-cysteine [BCYE(−)]. Colonies that can grow on BCYE agar, but not BCYE(−) agar, were considered presumptive *Legionella *species.

### 2.4. Sero- and Subgrouping of Isolates

*L. pneumophila* isolates were first serogrouped with a Latex agglutination test commercial kit (code DR0800; Oxoid; UK) according to the manufacturer's instructions. Further confirmation of serogroup was conducted with the Dresden panel of monoclonal antibodies [23].

### 2.5. Real-Time Quantitative PCR

Real-time PCR system (7500 Real time PCR system, Applied Biosystems; USA) and TaqMan *Legionella pneumophila* Detection Kits (Applied Biosystems P/N ED 000833; USA) were used for detection and quantification. All the samples were run with real-time PCR instrument using their DNA extracted from final concentrates using Clean Water DNA Extraction Kit (Applied Biosystems P/N ED 000849; USA) according to the manufacturer's instructions.

### 2.6. Antimicrobial Susceptibility Testing

Susceptibility testing was performed for 10 antimicrobials using *E*-tests with the lowest available MIC range. The tested antimicrobials were Moxifloxacin, Cefotaxime, Tigecycline, Clarithromycin, Rifampicin, Azithromycin, Erythromycin, Levofloxacin, Doxycycline, and Ciprofloxacin. Antimicrobial susceptibility testing was carried out as described by Bruin et al., 2012 [24]. In brief, isolates were recultured for 2-3 days at 35°C with increased humidity. Colonies were then suspended in sterile water to a concentration of 10^7^ CFU/mL and adjusted to 0.5 McFarland. A swab was then dipped in the suspension and used to inoculate a BCYE supplemented with ketoglutarate plates. The E test strip was then applied onto the swabbed surface, and the plates were then incubated at 35°C for 2 days with increased humidity. The MIC was read using a stereomicroscope from the scale of the strip at the point where the ellipse of growth inhibition intercepted the strip.

## 3. Results 

A total of 204 water samples were collected from different locations of residential water supply facilities in Kuwait. All samples were tested by the standard culture method and by RT-PCR for the detection of *L. pneumophila. *Out of the 204 water samples tested, only 48 samples were positive by standard culture method (23.5%), whereas 85 (41.7%) samples were positive by RT-PCR (Table 1). This higher detection capabilities of real-time PCR compared to the culture method have been previously reported [25–28].

The range (CFU/mL) of *Legionella* spp. and the prevalent serogroups isolated from water samples are shown in Table 2. Using the standard culture method, all positive samples showed count less than 10^4^ CFU per liter, the upper limit that represents a potential health risk to humans [5, 6]. Serogrouping of the 48 *Legionella* isolates is also shown in Table 2.* L. pneumophila *accounted for all the isolated *Legionella* species. Among the 46 *L. pneumophila* isolates, the majority of the isolates belonged to serogroup 3 (37 isolates), followed by serogroup 1 (6 isolates), serogroup 7 (1 isolate), serogroup 10 (1 isolate), and serogroup 4 (1 isolate). All six isolates of serogroup 1 were of the OLDA/Oxford subgroup.

The susceptibility of the *L. pneumophila* isolates (MIC's range mg/L) to 10 antimicrobials are shown in Table 3. In general, all isolates were inhibited by low concentrations of macrolides and fluoroquinolones. As an example, for serogroup 1, the minimum inhibitory concentration for the macrolides, MICs ranged from 0.19–0.25 for Erythromycin, 0.094–0.75 for Azithromycin, and 0.125–0.19 mg/L for Clarithromycin, respectively, while for the Fluoroquinolones MICs ranged from 0.38–0.5 *μ*g/mL for Ciprofloxacin, 0.25–0.38 mg/L for Levofloxacin, and 0.50–1.0 mg/L for Moxifloxacin, respectively. The MIC for Rifampicin was determined to be 0.008–0.016, 0.047–0.38 for Cefotaxime, and 2-3 mg/L for Tigecycline, and 2 for Doxycycline, respectively. A similar susceptibility pattern was also observed for serogroup 3. The antimicrobial Rifampicin was found to be the most active of the antimicrobials against all *L. pneumophila *serogroups.

## 4. Discussion

In this study, we examined the prevalence of *Legionella pneumophila* in the local residential water systems in the State of Kuwait. RT-PCR was carried out in parallel with the standard culture method in analyzing 204 water samples. Our results have shown that RT-PCR was more sensitive, less time consuming, and provides reproducible results making it more suitable as a screening assay to detect and quantify *L. pneumophila *in environmental water samples. However, PCR-based methods cannot differentiate between live and dead bacteria. Therefore, the culture method remains necessary for epidemiological comparison between clinical and environmental strains and to confirm an outbreak by PFGE analysis. Thus, quantitative real-time PCR would complement the reference standard culture method.

This study also showed the frequent isolation of *L. pneumophila* serogroup 3 as opposed to serogroup 1. Although the most frequent cause of legionnaires disease is *Legionella* serogroup 1, serogroup 3 has also been recently reported to cause community acquired and nosocomial pneumonia [29, 30]. Although the number of isolates was small in this study (*n* = 46), the predominance of *L. pneumophila* serogroup 3 from environmental samples may be useful in explaining the epidemiology of this serogroup in clinical and environmental outbreaks.

This study has also shown the antimicrobial susceptibility of all the isolate tested against antimicrobials often used to treat legionellosis. Macrolides are the most commonly used antimicrobial to treat legionellosis [31]. In particular, Rifampicin and the macrolides have demonstrated good activity against *L. pneumophila* serogroup 1 and 3. To our knowledge, this is the first survey on the prevalence and antimicrobial susceptibility of *Legionella *isolated from environmental water systems in residential facilities in the State of Kuwait. 

## 5. Conclusion

*Legionella* is a common pathogenic colonizer of water distribution systems and cooling towers leading to severe health risks and considerable legal and economic damage to businesses. Although, environmental *Legionella* monitoring is recommended in several countries, in Kuwait, environmental *Legionella* is not being monitored. The study confirms the presences of *L. pneumophila *serogroup 1 and 3 in Kuwait's domestic water systems with the most commonbeing* L. pneumophila *serogroup 3 (77.1%). The predominance of *L. pneumophila *serogroup 3 in water systems of residential facilities in Kuwait warrants further investigations to predict the risk that this serogroup plays in any future legionellosis outbreaks.

## Figures and Tables

**Table 1:** Comparison of culture method with real-time PCR for the detection of *Legionella* spp.

Water source	PCR^+^	Culture^+^	PCR^+^/culture^+^	PCR^+^/culture^−^	PCR^−^/culture^+^
Bathroom	54.9% (45/82)^a^	32.9% (27/82)	30.5% (25/82)	24.4% (20/82)	2.4% (2/82)
Kitchen	37.3% (19/51)	29.4% (15/51)	25.5% (13/51)	11.8% (6/51)	3.9% (2/51)
Water tanks	29.6% (21/71)	8.5% (6/71)	8.5% (6/71)	21.1% (15/71)	0% (0/71)

Total	41.7% (85/204)	23.5% (48/204)	22.1% (45/204)	20.1% (41/204)	2% (4/204)

^a^Number of positive samples/total number of samples.

**Table 2:** Range of *Legionella* cells in positive samples and prevalent serogroups.

Source (*n*)	Positive samples (*n*)	Range (CFU/L)	Total isolates	*Legionella* serogroups					
				Group 1	Group 3	Group 4	Group 7	Group 10	*L*. species
Bathroom (45)	PCR^+^/culture^+^ (25)	10–2250	27	3	21	0	0	1	2
	PCR^−^/culture^+^ (2)	20–130							
Kitchen (19)	PCR^+^/culture^+^ (13)	10–1250	15	3	10	1	1	0	0
	PCR^−^/culture^+^ (2)	10–30							
Tank (21)	PCR^+^/culture^+^ (6)	10–500	6	0	6	0	0	0	0
	PCR^−^/culture^+^ (0)								

**Table 3:** Antibiotics MICs for isolated *Legionella pneumophila *serogroups.

Antibiotics (mg/L)	Range (mg/L)	Serogroup 1 (*n* = 6)	Serogroup 3 (*n* = 37)	Serogroup 4 (*n* = 1)	Serogroup 7 (*n* = 1)	Serogroup 10 (*n* = 1)
Moxifloxacin	0.25–1	0.5–1	0.38–1	0.5	1.0	0.38
Cefotaxime	0.25–2	0.047–0.38	0.064–1	0.094	0.5	0.5
Tigecycline	1–16	2-3	2–4	4	2	3
Clarithromycin	0.064–1	0.125–0.19	0.125–0.25	0.125	0.125	0.19
Rifampicin	0.004–0.032	0.008–0.016	0.008–0.016	0.008	0.012	0.012
Azithromycin	0.032–8	0.094–0.75	0.047–0.19	0.125	0.094	0.38
Erythromycin	0.032–2	0.19–0.25	0.064–0.38	0.19	0.19	0.25
Levofloxacin	0.064–1	0.25–0.38	0.19–0.5	0.19	0.38	0.25
Doxycycline	1–8	2	1.5–3	3	2	2
Ciprofloxacin	0.008–1	0.38–0.5	0.38–1	0.5	0.5	0.38
